# Non-invasive reconstruction of dynamic myocardial transmembrane potential with graph-based total variation constraints

**DOI:** 10.1049/htl.2019.0065

**Published:** 2019-11-26

**Authors:** Shuting Xie, Linwei Wang, Heye Zhang, Huafeng Liu

**Affiliations:** 1 State Key Laboratory of Modern Optical Instrumentation, Department of Optical Engineering, Zhejiang University, Hangzhou 310027, People's Republic of China; 2 Computational Biomedicine Laboratory, Golisano College of Computing and Information Sciences, Rochester Institute of Technology, Rochester, NY 14623, USA; 3 School of Biomedical Engineering, Sun Yat-Sen University, Shenzhen 510006, People's Republic of China

**Keywords:** bioelectric potentials, graph theory, cardiology, diseases, phantoms, minimisation, medical signal processing, physiological models, electrocardiography, signal reconstruction, dynamic myocardial transmembrane potential, graph-based total variation constraints, electrophysiological activity, clinical disease prevention, surgical treatment, three-dimensional myocardium, heart disease diagnosis, myocardial ischemia, ectopic pacing, local similarity, dynamic TMP sequence, regularisation method, graph structure, heart node, infarct scar reconstruction, activation wavefront reconstruction, phantom experiments

## Abstract

Non-invasive reconstruction of electrophysiological activity in the heart is of great significance for clinical disease prevention and surgical treatment. The distribution of transmembrane potential (TMP) in three-dimensional myocardium can help us diagnose heart diseases such as myocardial ischemia and ectopic pacing. However, the problem of solving TMP is ill-posed, and appropriate constraints need to be added. The existing state-of-art method total variation minimisation only takes advantage of the local similarity in space, which has the problem of over-smoothing, and fails to take into account the relationship among frames in the dynamic TMP sequence. In this work, the authors introduce a novel regularisation method called graph-based total variation to make up for the above shortcomings. The graph structure takes the TMP value of a time sequence on each heart node as the criterion to establish the similarity relationship among the heart. Two sets of phantom experiments were set to verify the superiority of the proposed method over the traditional constraints: infarct scar reconstruction and activation wavefront reconstruction. In addition, experiments with ten real premature ventricular contractions patient data were used to demonstrate the accuracy of the authors’ method in clinical applications.

## 1 Introduction

Using electrophysiological imaging (ECGI) to depict the electrophysiological information within the heart has become a research hotspot for the diagnosis and therapy of heart disease. By selecting appropriate source and volume conductor model, one can reconstruct the potential distribution or the excitation propagation on the surface of the heart or in the three-dimensional (3D) ventricles, and then determine the location and extent of the lesion based on the anomalous characteristics that appeared [1, 2].

Common sources include transmembrane potential (TMP) [3], endo- and epicardial potential (EEP) [4], and activation time [5]. Among them, TMP was selected as the research target of this paper since it provides the most abundant physiological information and allows stronger prior information constraints [6]. However, it is ill-posed to reconstruct myocardial TMP from body surface potential (BSP) due to the following two points: (i) the dimensions of known quantities do not match those of unknown quantities (for accuracy, the number of unknown points on the heart is much larger than the number of surface leads). (ii) According to the Helmholtz's equivalent double-layer principle of the electromagnetic field, an infinite number of intramural solutions fit the same electrical field on the surface. Hence, researchers have made great efforts to add appropriate constraints on the solution space, called regularisation [7].

The most classic method is Tikhonov regularisation [8], which imposes a neighbourhood smoothing constraint and provides a solution with compromise accuracy. In recent years, combined with the sparsity and piece-wise smoothness of the potential on the heart, reconstruction methods based on sparse expression have been proposed, such as total variation (TV) minimisation [9]. These methods impose a constrain term with L1 norm form, which performs better than the L2 norm form in maintaining the steeply changing area of potential. However, they can only process one frame of data at the same time. If the whole sequence is to be solved, the computational time will increase linearly with the length of the sequence. The low rank and sparse constraint assume that the solution is composed of low-rank background and sparse foreground [10]. This method treats the solution of the whole sequence as a matrix, which takes into account the time dependence of the solution. However, singular value decomposition (SVD) in high-dimensional solution space in each iteration also makes the method computationally expensive.

With the rapid development of deep learning in the medical field, researchers try to solve the inverse problem of ECG by data-driven methods. Ghimire *et al.* trained a large number of simulation data to learn the general transformations of BSPs to TMP [11], and discussed the influence of different autoencoder architectures on the results [12]. However, there is no clinical method for directly measuring cardiac TMP values. Therefore, no real patient data participates in the training.

In order to make use of time dependence while avoiding frequent singular value decomposition, and taking into account the piecewise smoothness and sparsity in space, we propose a novel method, the graph-based TV reconstruction. This method improves the classical TV minimisation. We regard the records on each point in the sequence as a graph signal, making full use of the temporal and spatial correlations of the myocardial TMP distribution. Specific contributions are as follows:

(i) We construct a graph structure on the preliminary estimation of the solution which is based on the spatial surface smoothing hypothesis. This structure constructs a similarity relationship among the whole 3D myocardium, making full use of the underlying nonlocal features.
(ii) Different penalty weights are given to nodes with different similarities, which enhance intra-class consistency and inter-class differences.
(iii) The criterion for judging similarity is the Euclidean distance between TMP value vectors over a period of time at each node. Therefore, the temporal correlation among dynamic TMP sequence frames is taken into account. This makes the method more robust than that based on a single frame, and less affected by noise.
(iv) The mentioned method solves the whole sequence at the same time, avoiding the computational time increases linearly with the length of the sequence.
(v) The mentioned method is based on the spatio-temporal distribution characteristics of the solution, without the need for training based on large amounts of data.

## 2 Methodology

### 2.1 TMP imaging model

The forward relationship between TMPs and BSPs can be modelled as the following linear equation by applying the quasi-static approximation of electromagnetic theory [13]:

(1) $${\bf \Phi } = {\bf HU}\eqno\lpar 1\rpar $$

in which ${\bf \Phi } \in R^{m \times t}$ represents the *m*-lead BSPs of a length *t*, ${\bf H} \in R^{m \times n}$ is the transform matrix which contains the geometry and conductivity information in the heart-torso structures, obtained by finite element method (FEM) or boundary FEM (BEM). ${\bi U} \in R^{n \times t}$ is the *n*-dimensional TMPs of a length *t*. *t* represents the information of time dimension. The equation denotes the forward mapping relationship between dynamic TMP sequence and dynamic BSP sequence. To overcome the ill-posedness of this problem, we start from the characteristics of the spatio-temporal distribution of dynamic TMP sequence to find reasonable constraints for the solution.

**Fig. 1:**
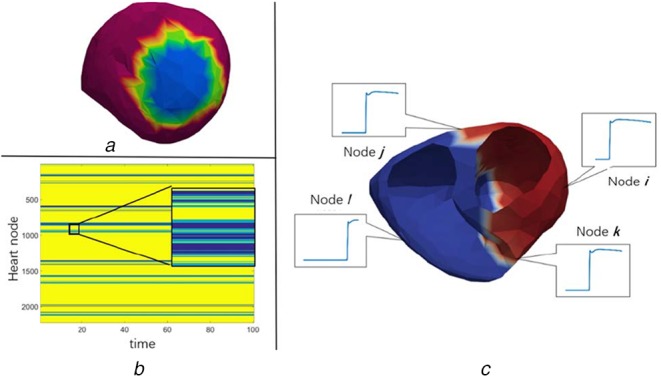
Piecewise smoothing and gradient sparse feature of the TMP distribution *a* TMP distribution of the 3D myocardium at a certain time (ischaemia) *b* Matrix ***U***∈*R*^(2223 × 100), the 2D version of (a) *c* Value on the different nodes of the heart

First, we considered the distribution of TMP on the myocardium during infarction or myocardial ischaemia cases. At the ST segment of the electrocardiogram (ECG), the ventricular cells are in a plateau phase in which all depolarisations are completed and sharp repolarisation has not yet begun. The potential distribution is flat, which corresponds to the condition of the normal heart. For infarcted or ischaemic cells, their action potentials are reduced due to the decreased or absent cell activity. This is prominent in a flat TMP distribution. There is a clear boundary between the normal area and the lesion area, as shown in Fig. 1.

For the case of the activation sequence, there are many nodes activated at the same time, but they are not necessarily concentrated near the same area. The TMP value changes for nodes activated at the same time are similar for a period of time. Fig. 1*c* shows this feature. This example is the spatial distribution of TMP at about 120 ms after the onset of pacing rhythm. The red part indicates high potential. The white line is the active wavefront and is annular in the myocardium. We can see that the nodes *j* and *k* have approximate values and may be considered adjacent in the graph, although they are separated in space.

The piecewise smoothing and gradient sparse feature of the TMP distribution prompts us to find a constraint to enhance the similarity between nodes in similar classes, and to distinguish between different classes of nodes. This makes the border clearer and can accurately indicate the lesion and the activation wavefronts.

### 2.2 Constraint regularisation

The TV method is the most classic sparse constraint method. Comparing Figs. 1*a* and *b*, we can see that in matrix ${\bi U}$, the same values are not simply clustered together. This is caused by the way the FEM models the heart. The adjacent nodes in the 3D space are not spatially adjacent in the 2D matrix ${\bi U}$ expanded by the serial number. The classic TV method uses adjacency matrix in 3D space to find spatial adjacent nodes and minimise the TV among them. However, this brings several problems that limit the improvement of precision: (i) The adjacency matrix of 3D space is the only prior to this problem, so it puts high requirement on the accuracy of cardiac modelling. (ii) It can only use the information between spatial adjacent nodes, similarities between those nodes which are not adjacent in space, but still belong to the same class are not available. (iii) It only considers the situation at a certain moment, without using the time correlation of dynamic sequences. This inspired us to propose a graph-based TV constraint.

#### 2.2.1 Neighbourhood-smoothness estimation

First, we apply a spatial neighbourhood-smoothing constraint to overcome the mathematical ill-posedness of the problem. This low-resolution solution $\hat{\bi U}$ will be used as the initial input for the subsequent optimisation algorithm.

(2) $$\hat{\bi U} = \left({{\bf H}^{\rm T}{\bf H} + \lambda {\bi L}^{\rm T}{\bi L}} \right)^{ - 1}{\bf H}^{\rm T}{\bf \Phi }\eqno\lpar 2\rpar $$

***L*** is the surface Laplacian operator, which utilises the spatial connection information of the nodes in the 3D heart model.

#### 2.2.2 Graph-based TV constraint

In recent years, graph-based regularisation methods have attracted much attention in image and manifold processing, for the graph structure extracts and enhance the self-similarity of the signal [14]. Similar ideas have also been applied to medical image processing [15, 16]. A graph structure contains three core constituent factors: node, edge and weight matrix ***W***. Adjacent nodes are connected by edges. Here judging whether adjacent or not is based on the similarity of the values on the nodes, not just the spatial connection which classical total variation takes as a priori. The degree of similarity of two nodes *i*, *j* is measured by the Euclidean distance:

(3) $$\ell = \left\Vert {u_i - u_j} \right\Vert _2\eqno\lpar 3\rpar $$

The vector ${\bi u}$ represents the value on one node, which is, in our case, a record of the TMP values overtime on a single node of the heart. A smaller $\ell $ indicates a high consistency between two nodes, meaning that they are more likely to be in the same state, e.g. both infarct cells or cells activated at close time. The k-nearest neighbour (KNN) search algorithm finds the distance for each pair of nodes, then returns the nearest *k* ones for each node, which are considered to be adjacent. The graph TV constraint can be written as

(4) $$\left\Vert {\nabla _G{\bi U}} \right\Vert _1 = \mathop \sum \limits_{i \in n} \left\Vert {\nabla _Gu_i} \right\Vert _1 = \mathop \sum \limits_{i \in n} \mathop \sum \limits_{\,j \in n_i} \sqrt {{\bi W}\left({i\comma \; {\rm \; }j} \right)} \left\Vert {u_i - u_j} \right\Vert _1\eqno\lpar 4\rpar $$

where *n* denotes the total number of nodes representing the heart. $n_i$ is a collection of all the neighbours of node *i*, the result from KNN. For a pair of adjacent nodes with a short edge (small $\ell $), we apply a large weight to minimise the gradient between them. For those nodes that have a large dissimilarity, we reduce the weight between them. Based on this, we choose the following form of weight matrix definition with parameter *σ* being the average distance of the connected nodes:

(5) $${\bi W}_{i\comma j} = \exp \left({ - \displaystyle{{\left\Vert {u_i - u_j} \right\Vert _2^2 } \over {\sigma ^2}}} \right)\eqno\lpar 5\rpar $$

### 2.3 Optimisation algorithm

Our reconstruction problem can be written as the following constraint minimisation form:

(6) $$\mathop {\min }\limits_{{\bi U} \in R^{n \times t}} \; \left\Vert {{\bf HU} - {\bf \Phi }} \right\Vert _F^2 + \mu \left\Vert {\nabla _G{\bi U}} \right\Vert _1\eqno\lpar 6\rpar $$

where ${\bf \Phi }$ represents the BSPs of a length *t*. ${\bf H}{\rm \; }$ is the transform matrix. ${\bi U}{\rm \; }$ is the transmembrane potentials of a length *t*. $\mu $ is a positive weighting parameter used to balance data fidelity term with regularisation term.

The problem (6) can be solved using forward–backward primal-dual method [17, 18]. This algorithm minimises a non-differentiable function by combining a gradient descent step (forward) with a proximal point step (backward). We split the target problem (6) into two sub-functions:

(7) $$h\left({\bi U} \right)= \left\Vert {{\bf HU} - {\bf \Phi }} \right\Vert _{\rm F}^2 \eqno\lpar 7\rpar $$

(8) $$g\left({L{\bi U}} \right)= \mu \left\Vert {\nabla _G{\bi U}} \right\Vert _1\eqno\lpar 8\rpar $$

$h\left({\bi U} \right)$ is the data fitting term with a Lipschitz continuous gradient

(9) $$\nabla h\left({\bi U} \right)= 2{\bi H}^{\rm T}\left({{\bf HU} - {\bf \Phi }} \right)\eqno\lpar 9\rpar $$

As for $g\left({L{\bi U}} \right)$, *L* is a linear operator, which in our case the graph gradient operator $\nabla _{\bi G}$. Since the L1 norm is non-differentiable, we calculate its proximal operator by the soft-thresholding method [19]

(10) $${\rm pro}{\rm x}_{\sigma g}\left(x \right)= {\rm sgn}\left(x \right)\cdot \max \left({\left\vert x \right\vert - \sigma \comma \; 0} \right)\eqno\lpar 10\rpar $$

(11) $${\rm pro}{\rm x}_{\sigma g^{\rm \ast }}\left(x \right)= x - \sigma {\rm pro}{\rm x}_{1/\sigma g}\left({\displaystyle{x \over \sigma }} \right)\eqno\lpar 11\rpar $$

**Fig. 2:**
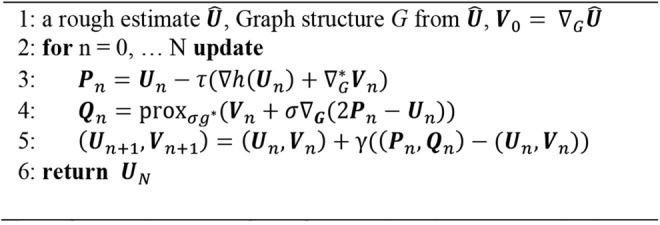
Algorithm 1: primal-dual for graph TV

By alternately solving the forward gradient and backward proximal problems, the algorithm finally converges to the final optimal solution. To ensure the convergence of the algorithm, the step size parameters should be chosen rigorously. The Lipschitz constant $\beta $ of $h\left({\bi U} \right)$ equals to $2\left\Vert {\bf H} \right\Vert _2$. We set $\tau = 1/\beta $, $\gamma = 0.99$. The algorithm converges under the condition that (see Fig. 2)

(12) $$\tau ^{ - 1} - \sigma \left\Vert L \right\Vert _2^2 \ge \displaystyle{\beta \over 2}\eqno\lpar 12\rpar $$

We have tested the reconstruction quality of different $\mu $ values within a certain range, for there are no mature ways to decide the best choice for this regularisation parameter. Specific details will be discussed in Section 4.1.

## 3 Experiments

We designed two sets of simulation experiments and one set of real patient experiments to verify the superiority and accuracy of our method. The ground truth and heart model of the simulation experiments are from the ‘Experimental Data and Geometric Analysis Repository’ (EDGAR) database [20]. This is an Internet-based archive of curated data that is freely distributed to the international research community for the application and validation of electrocardiographic imaging (ECGI) techniques.

### 3.1 Infarct scar reconstruction

In this section, we set the infarct nucleus locating at eight different segments to explore the performance of our method in reconstructing different infract locations. To test the robustness of the method to different noises, we add 5, 10, 15, 20, 25, 30 dB noise to the simulated BSP. We focus on comparing the performance between the graph-TV method and the classic TV method. The TV method we choose is Iteratively Re-weighted Minimisation of TV (IRTV) [10], which has been used to solve the myocardial TMP inverse problem.

**Fig. 3:**
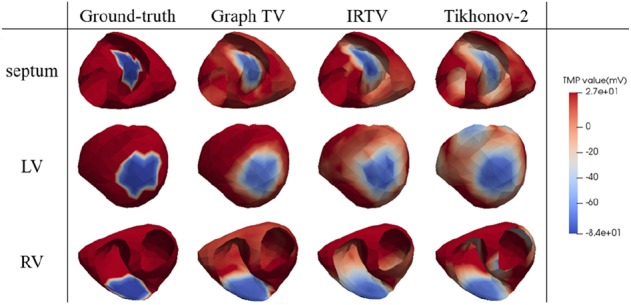
3D map of reconstructed TMP spatial distribution of infarct areas located in different regions with the body surface potential being disturbed by 20 dB noise

**Fig. 4:**
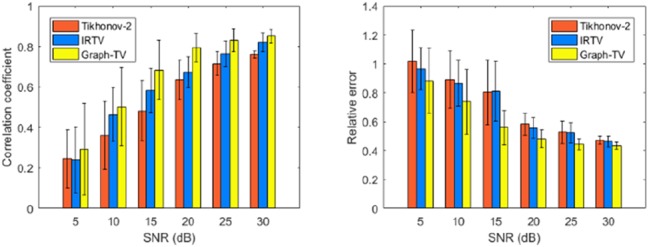
Comparison of CC and RE for three methods at different noise levels. The height of the column represents the average result value at a particular signal to noise ratio. Error bar shows the standard deviation among eight different segments *a* CC *b* RE

We set the TMP value of the infarct site to −84 mV and the healthy site to 27 mV as the reference ground truth, with sequence length to 100 ms. It can be found from Fig. 3 that the infarct area reconstructed by Graph-TV method has a higher consistency with the ground truth, with the scar shape being closer to the reference and the edge representation being more accurate. In contrast, the Tikhonov method provides an overly smooth solution, and its location has some artefacts. The IRTV method eliminates artefacts and provides a higher quality solution. Fig. 4 shows the numerical analysis results for 8 locations, total 48 cases when the body surface potentials were disturbed by noise in the range of 5–30 dB. Since our method considers the TMP distribution over the entire sequence, it provides better performance than the frame-by-frame calculation methods.

The accuracy of the method was evaluated by comparing the correlation coefficient (CC) and the relative error (RE) between the ground truth (subscript *t*) and the reconstructed TMP value (subscript *r*).

(13) $${\rm CC} = \displaystyle{{{\rm Cov}\left({{\bi x}_r\comma \; {\bi x}_t} \right)} \over {\sqrt {D\left({{\bi x}_r} \right)} \sqrt {D\left({{\bi x}_t} \right)} }}\eqno\lpar 13\rpar $$

(14) $${\rm RE} = \sqrt {\displaystyle{{\mathop \sum \nolimits_{i = 1}^N {\left({{\bi x}_{r_i} - {\bi x}_{t_i}} \right)}^2} \over {\mathop \sum \nolimits_{i = 1}^N {\left({{\bi x}_{t_i}} \right)}^2}}} \eqno\lpar 14\rpar $$

### 3.2 Activation wavefronts reconstruction

This part of the experiment was designed to verify the accuracy of our method in reconstructing the activation propagation sequence. Eight ventricular pacing transmembrane potentials were interpolated on the coarse tetrahedral mesh. The depolarisation phase of pacing can reveal the abnormality in the conduction pathway, which is helpful in locating the ectopic site or the underlying pathology.

**Fig. 5:**
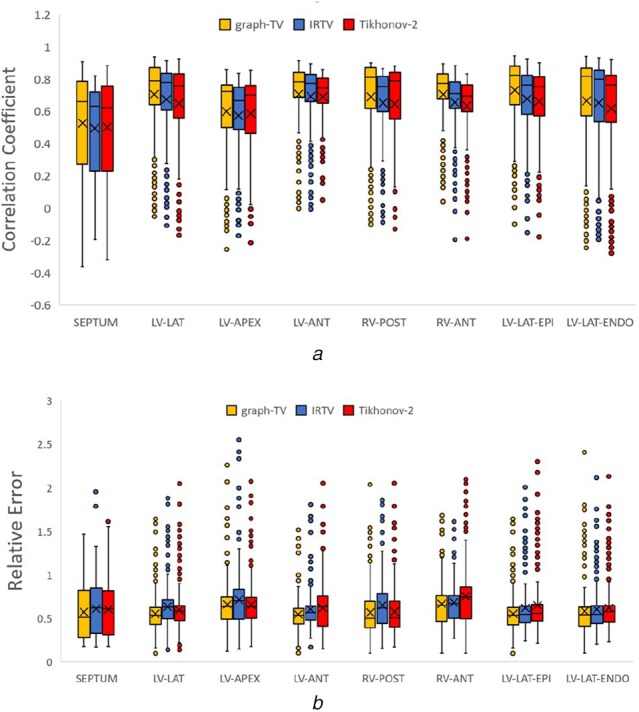
Comparison of CC and RE for three methods at different pacing sites *a* CC *b* RE

In the initial phase of pacing, the Tikhonov regularisation method is almost impossible to indicate the location of the pacing site. The L1-norm based sparse constraint makes the IRTV method perform better at the initial and final phases of the pacing. However, the position of the propagation wavefront cannot be accurately captured. The result of Graph-TV method has the highest concordance to reference ground truth during the whole process of propagation, with the closest wavefront shape. Fig. 5 shows the statistical results of the reconstruction at different pacing sites. Each sequence length is 150–250 ms. Outliers often appear at the beginning and end of the sequence. In this time period, the number of activated nodes (or inactivated nodes) is small, and accurate reconstruction is more difficult than others. The results of reconstruction at the septum are worse than those at other locations, for this location hides the deepest from the body surface.

**Fig. 6:**
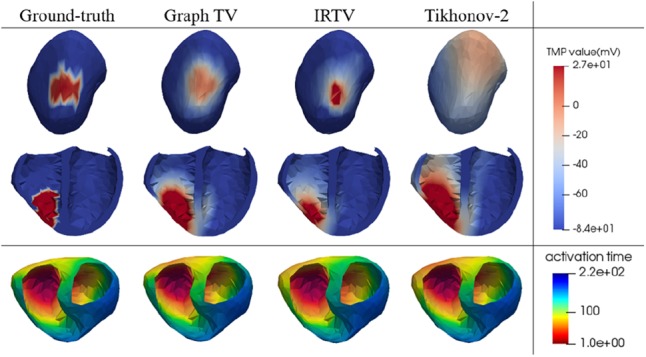
3D map of reconstructed TMP wavefront and activation time maps for pacing at RV-posterior

As another property describing the process of excitement propagation, the activation time map is used to visually indicate the order in which cardiomyocytes are activated. The earliest point of the activation time map is considered as the beginning point of pacing. In the case of Fig. 6, the location errors of listed three methods are 15.63 mm (Tikhonov-2), 9.95 mm (IRTV) and 7.44 mm (Graph-TV).

### 3.3 Real patient experiment

**Fig. 7:**
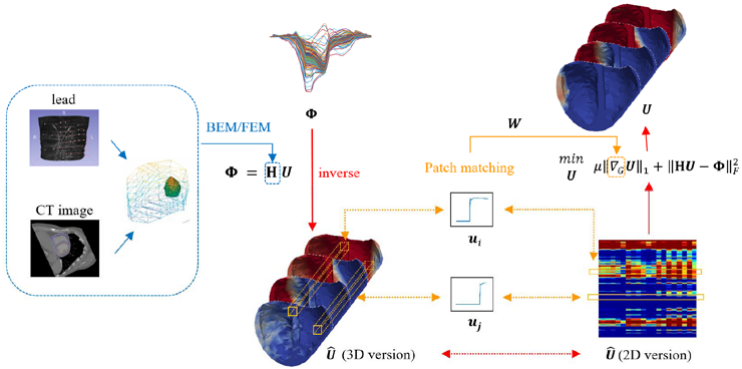
Illustration of the proposed graph-based TV reconstruction method. The solid red line represents the main flow. The blue part is the establishment of the **H** matrix. The yellow part is the creation of the graph. The dashed line of the double arrow shows the two parts are equivalent

In this section, we collected the data of ten premature ventricular contractions (PVCs) patients, and to non-invasively locate the lesion by the mentioned three methods to explore the potential of the methods in clinical application. We locate the pacing site by reconstructing the high-potential area in the early stages of pacing. The 64-lead ECG recorded the distribution of body surface potentials overtime during arrhythmia with a sampling frequency of 2 kHz, i.e. ${\bf \Phi }$, in our problem. The patient wears lead electrodes for a CT scan, whereby the position of the lead on the torso can be obtained to establish a torso model. We sliced the CT image containing the relative position of the heart-torso along the short axis of the heart, then labelled the contours of the epicardium, ventricular and right ventricular outflow tracts to obtain a 3D cardiac model. Finally, the two models are registered in the same coordinate system, and the anisotropic conduction information is combined to obtain a personalised TMP-BSP transfer matrix **H**. The blue part of Fig. 7 shows this process. The Ensite3000 diagnostic results of invasive surgery records are considered as the gold standard to verify the accuracy of non-invasive reconstruction results. Nine cases of the ectopic pacing site were located in the right ventricular outflow tract and one in the left ventricular apex.

**Fig. 8:**
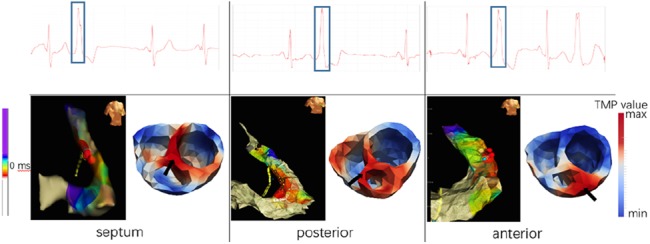
Clinical Ensite3000 diagnosis and graph-TV-based electrophysiological imaging diagnosis of three real PVC patients with ectopic pacing site located at RVOT, respect to case 1,2,3 in Table 1. In Ensite3000, the colour from red to blue indicates the active timing, and the torso in the upper right corner represents the orientation of the current model

The occurrence of PVC is usually accompanied by a large and deformed QRS wave, hence we select this segment for reconstruction. Table 1 lists the clinical diagnosis and reconstruction results of these ten patients. Fig. 8 shows the 37th lead ECG, the Ensite3000 diagnostic screenshot and the distribution of TMP on the 3D heart model of cases 1, 2 and 3. We reconstructed the QRS part (about 140–180 ms) and selected the early time nodes for 3D display. The high level (red) indicates the location of the pacing. Combined with Table 1, it can be seen that for patients with complete data collection (with CT scan data with 64 lead positions, so that a personalised cardiac torso model can be established), the proposed method can accurately determine the location of the pacemaker. For cases where CT images were not acquired, we used a general model, which made the reconstruction results of the two cases slightly offset (see cases 7 and 10). The personalised model contains patient-specific geometric location information and has an important impact on the quality of the reconstruction results. Experiments have shown that our method can clearly indicate the boundary between the active site and the resting site, so as to accurately locate the lesion.

**Table 1:** 10 cases of real patient data and diagnostic results

Case	Gender	Age	CT	PVC origin	Graph-TV result	IRTV result	Tikhonov-2 result
1	F	32	√	RVOT septum	RVOT septum	RVOT septum	RVOT septum
2	F	45	√	RVOT posterior	RVOT posterior	RVOT posterior	RVOT posterior
3	F	57	√	RVOT anterior	RVOT anterior	RVOT anterior	**RVOT free wall**
4	M	33	√	RVOT posterior	RVOT posterior	RVOT posterior	RVOT posterior
5	M	52	—	LV apex	LV apex	LV apex	LV apex
6	F	58	√	RVOT anterior	RVOT anterior	**RVOT septum**	RVOT anterior
7	F	67	—	RVOT posterior	**RVOT septum**	**RVOT septum**	**RVOT septum**
8	M	35	√	RVOT anterior	RVOT anterior	RVOT anterior	RVOT anterior
9	F	53	—	RVOT posterior	RVOT posterior	RVOT posterior	RVOT posterior
10	F	58	—	RVOT posterior	**RVOT septum**	**RVOT septum**	**RVOT septum**

## 4 Discussion

### 4.1 Parameter setting

In our proposed optimisation algorithm, the parameters *k* and *μ* are the two most critical parameters affecting the final result. The *k* value affects the accuracy of the graph structure, while *μ* is an important factor that weighs the data fidelity and sparse constraints.

**Fig. 9:**
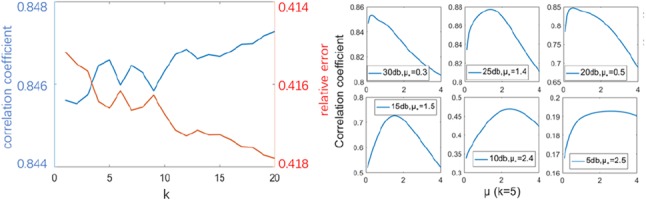
Parameters test *a* Effect of different *k* values on reconstruction results *b* Optimal *μ* value test at different SNR levels. *k* is fixed at 5

We first determine the value of *k* and then test the effect of different *μ* values based on a fixed *k*. After test we found that the value of *k* has little effect on the result compared to the value of *μ*. Fig. 9*a* shows that the gap between CC and RE is less than 0.005 when *k* changes from 0 to 20. Considering that a large *k* value will produce a significant oscillation near the optimal solution, while a too small *k* increases the calculation time, we finally choose *k* = 5 as our experimental parameter. Fig. 9*b* shows that the optimal value of *μ* increases as the noise level increases. This is in line with the general denoising problem we are familiar with.

### 4.2 Computing time

Here we discuss the computational time cost of the three methods mentioned. Table 2 shows the results of one set of activation wavefronts reconstruction experiments. The pacing site was located at LV-APEX, with a sequence length of 221 ms. The number of mesh nodes of the heart model is 2223. The experimental environment is MATLAB R2016a, with a 3.2 GHz processor and 16 GB RAM.

**Table 2:** Comparison of the computing time for three methods

	Tikhonov-2, s	IRTV, s	Graph-TV, s
single frame	3.7	6.2	4.3
sequence	7.8	542.9	14.2

The Tikhonov method takes the shortest time because it does not require iterative computation. The calculation of both single frame and sequence involve one singular-value decomposition. IRTV is an iterative algorithm, and the regularisation parameter is adjusted in each iteration to converge to the optimal solution. Each frame is computed independently, so the time-consuming increases almost linearly. Graph TV is an iterative algorithm, which converges to the optimal solution by solving the primal and dual problems alternately. Because the algorithm can solve the whole sequence at the same time, it has a great advantage in speed compared with the IRTV algorithm.

## 5 Conclusion

This paper proposes a graph-based TV constraint to solve the inverse problem of ECG imaging. Simulation experiments show that the proposed method has higher reconstruction quality than the Tikhonov method based on L2-norm and the classical TV method based on spatial adjacency constraints. Real patient experiments also illustrate the accuracy of the method of clinical application. Compared to the frame-by-frame reconstruction method, the sequence-based solution overcomes the disadvantage that the computation time increases linearly with the sequence length. Due to the lack of sufficient training data, this work has not been compared with the deep learning method for the time being. In the future, we will consider combining deep learning and traditional optimisation methods to improve the performance of TMP reconstruction in a data-driven manner.

## 6 Funding and declaration of interests

This work is supported in part by the National Natural Science Foundation of China (No: U1809204, 61525106, 61427807, 61701436), by the National Key Technology Research and Development Program of China (No: 2017YFE0104000, 2016YFC1300302), and by Shenzhen Innovation Funding (No: JCYJ20170818164343304, JCYJ20170816172431715).
